# Causal Inference between Rheumatoid Arthritis and Breast Cancer in East Asian and European Population: A Two-Sample Mendelian Randomization

**DOI:** 10.3390/cancers12113272

**Published:** 2020-11-05

**Authors:** Choonghyun Ahn, Sangjun Lee, Sue K. Park

**Affiliations:** 1 Department of Preventive Medicine, Seoul National University College of Medicine, Seoul 03080, Korea; ahnchoonghyun@gmail.com (C.A.); sjunlee@snu.ac.kr (S.L.); 2 Department of Biomedical Science, Seoul National University Graduate School, Seoul 03080, Korea; 3 Tokyo University Hospital, Tokyo 1130033, Japan; 4 Cancer Research Institute, Seoul National University, Seoul 03080, Korea; 5 Convergence Graduate Program in Innovative Medicine Science, Seoul National University College of Medicine, Seoul 03080, Korea

**Keywords:** breast cancer, rheumatoid arthritis, genetics, Mendelian randomization, causal inference

## Abstract

**Simple Summary:**

Rheumatoid arthritis (RA) is one of the chronic autoimmune diseases that affects about 0.5 to 1.0% of the general population worldwide. The main symptom of RA is the destruction of the synovial joint, leading to a reduced quality of life and increased mortality. RA may be accompanied by several comorbidities, on which several studies have been conducted on the association between RA and breast cancer. However, the association between RA and breast cancer has shown different directions and has not been clearly established. In this study, we tried to determine whether RA had a causal effect on breast cancer using Mendelian randomization (MR) analysis, but causal evidence was not found. Therefore, additional studies are needed to determine whether RA patients are at high risk of breast cancer, based on large-scale cohorts to validate these results.

**Abstract:**

Previous studies have been reported that the association between rheumatoid arthritis (RA) and breast cancer remains inconclusive. A two-sample Mendelian randomization (MR) analysis can reveal the potential causal association between exposure and outcome. A two-sample MR analysis using the penalized robust inverse variance weighted (PRIVW) method was performed to analyze the association between RA and breast cancer risk based on the summary statistics of six genome-wide association studies (GWAS) targeting RA in an East Asian population along with summary statistics of the BioBank Japan (BBJ), Breast Cancer Association Consortium (BCAC), and Consortium of Investigators of Modifiers of BRCA1/2 (CIMBA) targeting breast cancer. We found that the direction of the effect of RA on breast cancer varied among GWAS-summary data from BBJ, BCAC, and CIMBA. Significant horizontal pleiotropy based on a penalized robust MR-Egger regression was observed only for BBJ and CIMBA BRCA2 carriers. As the results of the two-sample MR analyses were inconsistent, the causal association between RA and breast cancer was inconclusive. The biological mechanisms explaining the relationship between RA and breast cancer were unclear in Asian as well as in Caucasians. Further studies using large-scale patient cohorts are required for the validation of these results.

## 1. Introduction

To date, the association between rheumatoid arthritis (RA) and cancer risk is unclear. The association of RA with some type of cancers, such as lymphoma and skin cancer, has been reported [1,2,3]. A meta-analysis conducted to determine the association between RA and breast cancer risk yielded inconclusive results [4]. Previous studies have suggested that the direction of RA risk effect may vary in different populations [4]. The risk of breast cancer was found to be consistently decreased in Caucasian RA patients [5,6,7,8,9], whereas it was increased in non-Caucasian RA patients [10,11,12]. Among the studies conducted on Caucasians, hospital-based studies showed a decreased risk of breast cancer, whereas population-based studies did not confirm risk reduction [4]. 

Although the underlying biological mechanism explaining the association between RA and cancer remains unclear, the biological pathways associated with the development and prognosis of breast cancer and RA share several components [13,14,15,16,17,18]. One of the possibilities of development of breast cancer in RA patients is though immunosuppressive therapy [19,20,21,22]. Immune suppression is known to play a role in the incidence and progression of breast cancer [23,24]. These findings suggest that the treatment options for RA patients may play a role in the development of breast cancer.

Several studies conducted to determine the association between breast cancer and RA reported inter-racial differences in their results. This may be due to differences in the incidence of breast cancer (a lower incidence and higher mortality rates in Asians than in Caucasians) [25] and differences in genetic structure and linkage disequilibrium between the two populations [26]. Another reason for the observed differences may be the insufficient consideration of internal validity and causality due to limitations in the study methods, such as hospital-based case-control study, cross-sectional study design, short-term follow-up in cohort studies, or insufficient control of confounders in observational studies, and the relatively small number of studies conducted in Asian and the small sample size of each Asian study. 

The Mendelian randomization (MR) method may be a useful tool to determine the association between diseases and exposure from independent observational studies based on the concept of solving the temporal relation in causality by using the germline variants as instrumental variables (IVs) to shift the exposure time of the main risk factor to the subject’s birth time or earlier [27]. 

Summary statistics of a genome-wide association studies (GWAS) conducted using the Biobank Japan (BBJ) [28], Breast Cancer Association Consortium (BCAC) [3,29], and Consortium of Investigators of Modifiers of BRCA1/2 (CIMBA) [30,31] are available to the public. The purpose of our study was to determine the association between RA and breast cancer in East Asian and European populations using the summary results of the BBJ, BCAC, CIMBA, and six GWASs targeting RA in the East Asian population.

## 2. Results

### 2.1. Two-Sample MR Analysis

The summary statistics including *p*-values, beta coefficients, standard errors (SEs), risk alleles, and risk allele frequencies for the association between single nucleotide polymorphisms (SNPs) and RA in previous GWAS papers and for the association between SNPs and breast cancer risk in the public GWAS summary statistics from the BBJ, BCAC, and CIMBA are shown in Tables S1 and S2. The two-sample MR analysis conducted using the BBJ data and the BCAC and CIMBA data, included 24 and 25 SNPs associated with RA, respectively. One SNP (rs1600249) was excluded due to an inconsistent minor allele frequency.

**Figure 1:**
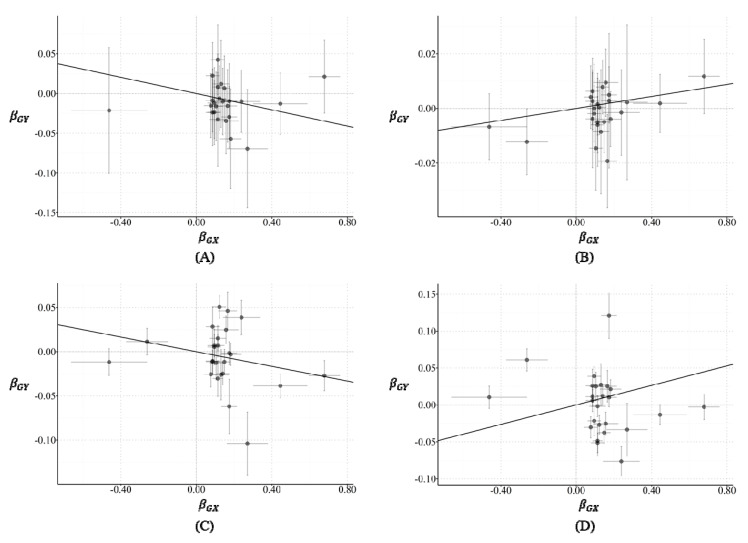
Scatter plots of the estimated effects of single nucleotide polymorphisms (SNPs) on rheumatoid arthritis (RA) against the estimated effects of SNPs on the risk of breast cancer. (**A**–**D**) are based on genome-wide association study (GWAS)-summary statistics for breast cancer in BBJ, BCAC, CIMBA-BRCA1 carriers, and CIMBA-BRCA2 carriers, respectively. *β_GX_* is calculated to estimate SNPs-RA association, and *β_GY_* is calculated to estimate SNPs-breast cancer association. The slopes of the lines are the estimated causal effects of RA on the risk of breast cancer, estimated using penalized robust inverse variance weighted (PRIVW) method.

Results of the penalized robust inverse variance weighted (PRIVW) analysis for causal inference of RA on breast cancer risk in two-sample MR are shown in Table 1. Figure 1 shows scatter plots of the estimated effects of SNPs on RA against the estimated effects of SNPs on the risk of breast cancer. Figure S1 presents the estimated causal change in standard deviations with a 95% confidence interval (CI) by forest plots. Furthermore, the intercept results for the MR Egger regression test for pleiotropy evaluation are shown in Table 2. RA significantly decreased the risk of breast cancer (odds ratio (OR): 0.95, 95% CI: 0.91–0.99) in the BBJ GWAS-summary data, whereas it significantly increased the risk of breast cancer (OR: 1.01, 95% CI: 1.00–1.03) in the BCAC GWAS-summary data. In BRCA1 carriers from CIMBA, RA showed a statistically significant decrease in breast cancer risk (OR: 0.96, 95% CI: 0.95–0.97). On the other hand, in BRCA2 carriers from CIMBA, RA showed an increased risk of breast cancer, despite a statistically insignificant result (OR: 1.07, 95% CI: 0.99–1.15).

### 2.2. Horizontal Pleiotropy 

No Significant horizontal pleiotropy based on PRIVW method was observed in overall breast cancer and hereditary breast cancer in BCAC and BRCA1 carriers. *P*-values for the intercept terms in penalized robust MR Egger regression were 0.31, 0.59, 0.04, and ≤0.001 for BCAC, CIMBA BRCA1 carriers, BBJ, and CIMBA BRCA2 carriers, respectively.

### 2.3. Funnel Plot

Figure S2 shows the results of funnel plot asymmetry displaying the estimates of precision (1/SE) and Wald ratios for each SNP.

## 3. Discussion

In this study, we used two-sample MR using PRIVW analysis to determine the association between RA and breast cancer using six GWAS targeting RA in an East Asian population and the summary results from the BBJ project, BCAC, and CIMBA targeting breast cancer. A previous study suggested the possibility that the direction of the effect of RA on breast cancer may vary in different populations [5,6,7,8,9,10,11,12]. The direction of the effect of RA on breast cancer in this study was consistent with the findings of previous epidemiological studies conducted on Caucasians [5,6,7,8,9]. On the contrary, previous studies targeting non-Caucasian populations had relatively small sample sizes, and the direction of the effect of RA on breast cancer in Asian populations was inconclusive. 

To confirm the association between RA and breast cancer, we focused on GWAS conducted in both the East Asian and European populations. Although the results of the two-sample MR analysis based on the BBJ, BCAC, and BRCA1 carriers from CIMBA were statistically significant, these findings were inconsistent with the GWAS summary statistics. The direction of association between RA and breast cancer seems to differ among Western and Asian populations, and there may be several possible explanations for this. First, the causal association between RA and breast cancer remains inconclusive due to the inconsistency of results among different databases. The other explanation is that our analysis only investigated the differences in the causal association between RA and breast cancer between Western (BCAC) and Asian ancestries (BBJ). Previous meta-analyses conducted to determine the association between RA and breast cancer risk found that RA increased the risk of breast cancer in non-Caucasians, whereas it decreased the risk in Caucasians [4]. In this meta-analysis, the ethnic differences of the association were suggested due to the difference in genetic predisposition [4]. Our study may also suggest that the different direction of causal association between Caucasians and non-Caucasians was due to the variation of genetic predisposition among different ethnicities, even though the direction of the effect of RA on breast cancer in our study differed from that of the previous meta-analysis [4]. As a result, MR analysis can be a useful tool that can give a causal explanation for ethnic differences due to genetic predisposition.

In the BRCA2 carriers from CIMBA, as the horizontal pleiotropy was significant and the PRIVW result was insignificant, confounders were not completely excluded, thus, the causality could not be investigated.

To investigate whether RA-associated SNPs were also associated with other phenotypes or not, we reviewed RA-associated SNPs in the GWAS catalog [32]. Table S3 shows that most of the RA-associated SNPs were only associated with RA, according to the GWAS catalog. Even though some of the RA-associated SNPs were associated with other phenotypes, other phenotypes were a type of autoimmune disease or were not considered as potential confounding factors. Nevertheless, based on the statistics of horizontal pleiotropy, there is a possibility that some genes associated with RA and breast cancer can overlap in the East Asian population and BRCA2 carriers.

The MR result of BRCA1 carriers from CIMBA suggested that RA is associated with a reduced risk of breast cancer, although most of the participants were Caucasian. Breast cancer from the BRCA1 carriers is a kind of early-onset, and many BRCA1 mutations cause breast cancer before age 45 [33]. Therefore, breast cancer from BRCA1 carriers is a type of premenopausal breast cancer and is associated with Triple-negative breast cancer (TNBC) which is defined as a subset of breast cancer with the lack of expression of estrogen receptor (ER), progesterone receptor (PR), and HER2. [34]. On the other hand, the onset age of breast cancer from BRCA2 carriers is 10 years older than that of breast cancer from BRCA1 carriers [33] and is highly associated with estrogen receptor (ER) positive breast cancer [34]. Moreover, in previous studies, the latency of RA was within five years [35], and the early age of RA diagnosis was defined as under 50 years [8]. Given that the age of breast cancer incidence is around 50 years of age [33], the incidence of RA should be at least 40 years of age [6]. Therefore, the MR results from the selection of genetic variants from the Asian population in our study, as expected, suggested that RA reduces the risk of breast cancer within participants from BRCA1 carriers, which mostly consists of Caucasians. As a result of MR analysis based on CIMBA-BRCA1 carriers, MR analysis can be also considered as a tool to determine the causality of different associations by genetic variations according to ethnic group.

Although the exact biological mechanism for the increased or decreased risk of breast cancer in RA patients is still unclear, several biological pathways may play a role. The mammalian target of rapamycin, which is known to be involved in the development of breast cancer [13] is known to be associated with the development and progression of RA [14,15]. MicroRNA-125, which is suspected to be associated with the development of RA [16] is known to be associated with the prognosis and development of breast cancer [17,18]. In addition, RA is an autoimmune disease and the treatment option usually includes immune suppression [36,37,38]. Immune escape is known to play a role in cancer incidence and prognosis, including breast cancer [39,40,41,42], and it is possible that the cytotoxic treatment in RA patients affects the oncogenic progression.

The incidence pattern of breast cancer in the Asian population is known to be significantly different from that of western populations [43], suggesting that the genetic patterns affecting breast cancer incidence may differ in the East Asian and western population. The difference in dosage and regimen of RA treatment between western and East Asian populations possibly caused the inconsistency in the direction of association [44]. Carcinoma-associated fibroblasts related to autoimmune diseases and cancer progression may play a different role in the Asian and western populations [4]. The results of this study were consistent with those of previous studies determining the association between RA and breast cancer risk in Asian populations. The inconsistency in the direction of association between RA and breast cancer in different populations may be related to the gene-environment interactions due to immune suppression and stimulation to adjust the risk of breast cancer.

There are some limitations to our results that need to be considered. Even though two-sample MR can be performed when the exposure of interest and the outcome are not simultaneously measured within one dataset, full datasets such as large-scale patient cohorts such as discovery and external validation sets are needed for a comprehensive understanding of causality, considering potential confounding factors. In other words, two-sample MR analysis is an indirect observation as compared to large-scale epidemiological studies, such as patient cohort analysis. The consistency of our results should be confirmed from various data sources. Moreover, not all genome-wide significant SNPs that predicted RA were available in the breast cancer GWAS we used. In addition, we included the results of a GWAS targeting RA in an East Asian population, but we used the summary results of the BCAC and CIMBA targeting breast cancer in European populations. Although summary statistics of the SNPs significantly associated with the exposure are required in MR analysis, BCAC and CIMBA have not opened the GWAS summary statistics to the public yet. Although there is a possibility that genome-wide significant SNPs associated with RA can differ according to the onset of RA, our study could not consider the variation of the RA onset. Since SNPs were obtained from six previous GWAS studies, we had a difficulty obtaining the information on the onset of RA.

## 4. Materials and Methods

### 4.1. Study Population

The summary statistics of GWAS for RA were extracted from six studies in the Korean and Japanese population [45,46,47,48,49,50]. Details of the quality control, imputation, and GWAS for each study have been previously described elsewhere [45,46,47,48,49,50].

Briefly, the study of Freudenberg et al. (2011) performed GWAS on the Korean population to identify susceptibility loci for RA, based on 801 RA cases and 757 controls for 441,398 SNPs. Among them, 79 SNPs were replicated by an independent European population, consisting of 718 RA cases and 719 controls [45]. The genome-wide significant *p*-value of SNPs was 5 × 10^−8^ in the discovery dataset. The study of Hu et al. (2011), also conducted GWAS on Koreans with 100 RA cases and 600 controls for 300,909 SNPs. Based on an independent case-control sample consisting of 578 RA cases and 711 controls, replication analysis was performed. A total of eight SNPs were selected with genome-wide significant *p*-value 1 × 10^−5^ [46]. The study of Kochi et al. (2010) performed GWAS in Japanese with 7069 RA cases and 20,727 controls for over 550,000 SNPs. Genome-wide significant *p*-value of SNPs was 5 × 10^−8^ [47]. The study of Myouzen et al. (2012) implemented GWAS with 7907 RA cases and 35,362 controls in the Japanese population. A landmark SNP was selected with *p*-values from 5 × 10^−8^ to 5 × 10^−5^ [48]. The study of Okada et al. (2012) conducted GWAS with 4074 RA cases and 16,891 controls for 1,948,139 SNPs. Replication analysis was also performed by an independent sample with 5277 RA cases and 21,684 controls. Genome-wide significant *p*-value of SNPs was 5 × 10^−8^ [49]. The study of Terao et al. (2011) performed GWAS with 1247 RA cases and 1486 controls for 277,420 SNPs. Replication analysis was conducted based on two independent samples consisting of 1865 RA cases and 1623 controls, and 2303 cases and 3380 controls. SNPs with a *p*-value < 1 × 10^−3^ were selected as candidates for further studies [50]. Five of the six summary statistics of GWAS for RA were obtained from the results of a meta-analysis, and the study of Kochi et al. (2010) was only based on a single study. All studies based on the meta-analysis did not report whether the meta-analysis was performed with a fixed or random-effect model. However, as we calculated effect sizes and confidence intervals of each SNP, all studies using meta-analysis approach were performed with fix effect model.

GWAS was conducted from BBJ, based on about 159,000 participants of Japanese ancestry [28]. Among participants, individuals in the GWAS for breast cancer consisted of 5552 cases and 89,731 controls. The processes of quality control, imputation, GWAS, and ethical approval in BBJ have been previously described in more detail elsewhere [51,52]. Genotyping from individuals of BBJ was performed based on either the Illumina Human Omni Express Exome BeadChip or a combination of the Illumina Human Omni Express and Human Exome BeadChips. Imputation was conducted based on the combination of whole-genome sequencing data from BBJ1K [53] and the 1000 Genomes Project. BBJ was approved by the ethics committees of RIKEN Center for Integrative Medical Sciences and the Institute of Medical Sciences, the University of Tokyo.

GWAS from the BCAC was conducted with the largest breast cancer. The processes of genotyping, quality control, and imputation in BCAC are already provided in more details elsewhere [3]. The BCAC consists of 122,977 cases and 105,974 controls of European ancestry for 11,792,543 SNPs. Among participants in BCAC, 46,785 cases and 42,892 controls from 211,155 SNPs were genotyped using the Illumina iSelect genotyping array (ICOGS) and 61,262 cases and 45,494 controls from 570,000 SNPs were genotyped by the OncoArray from Illumina (Illumina, San Diego, CA, USA). Genotyping data from all individuals of BCAC were imputed based on the 1000 Genomes Project Phase 3 v5 EUR reference panel [54]. With adjustment for country and top principal components, logistic regression was conducted to estimate ORs per allele.

The CIMBA consists of 15,252 BRCA1 and 8211 BRCA2 carriers, of whom 12,127 participants with breast cancer (7797 BRCA1 and 4330 BRCA2 carriers). The procedures of genotyping and quality control have already been provided in more details elsewhere [30,31]. Shortly, samples with a genotyping call rate <95% were excluded, considering excessive outlier of heterozygosity. Participants who was not female or had ambiguous sex or duplicates were also excluded. A total of 570,000 SNPs was genotyped using the OncoArray BeadChip from Illumina (Illumina, San Diego, CA, USA). Genotyping data from all individuals were imputed based on the 1000 Genomes Project Phase 3 v5 EUR reference panel [54]. After imputation, SNPs with imputation R^2^ ≤0.3 and minor allele frequency (MAF) ≤0.005 were excluded in further analyses. SNPs with a call rate under 95% were also removed.

### 4.2. Exposure and Outcome

SNPs associated with RA in the East Asian population were identified from the summary statistics of six GWAS [45,46,47,48,49,50]. SNPs with known risk alleles, beta coefficients, and SEs were included in the study. We selected a total of 25 SNPs as IVs with *p*-values below 1 × 10^−5^, considering statistically significant. The beta coefficients and SEs of these SNPs were searched from the summary results of the following GWAS data sources: BBJ [28], BCAC [29], and CIMBA [30]. SNPs with inconsistent minor alleles from different GWASs were excluded from the analysis. The overall workflow for the data extraction and analysis is shown in Figures S3 and S4.

### 4.3. Statistical Analysis

**Figure 2:**
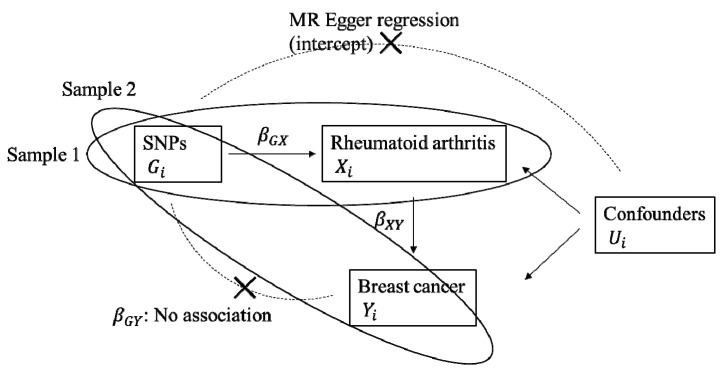
Two-sample Mendelian randomization testing the causal effect of rheumatoid arthritis (RA) on the risk of breast cancer. Estimates of the single nucleotide polymorphisms (SNPs)-RA association (*β_GX_*) were calculated in ‘Sample 1’ and the estimates of the SNPs-breast cancer association (*β_GY_*) were calculated in ‘Sample 2’. Finally, the estimates of SNPs are combined using the two-sample MR approaches, using penalized robust inverse variance weighted analysis (*β_IV_*) to confirm an overall causal estimate of RA on breast cancer risk and penalized robust MR-Egger regression with intercept to evaluate the possibility of pleiotropy. *G_i_,* SNPs; *X_i_,* Rheumatoid arthritis; *U_i_,* Confounders; *Y_i_,* Breast cancer.

MR analysis is a method to measure the degree to which the genetically predicted exposure (*X_i_*) of interest has a causal effect (*β_GX_*) on the outcome (*Y_i_*) by genetic variants as IVs, which are strongly associated with exposure (Figure 2). In this study, to evaluate the causality of genetically predicted RA on the increased risk of breast cancer, we expected *β_GX_* for RA as exposure, but not *β_XY_* for breast cancer as exposure, to be significantly greater than zero.

A recent MR analysis available for GWAS-summarized data was used to estimate the associations of genetic variants with the risk factors or the outcomes, calculated by beta coefficients and SEs [55]. The estimation of the causal effect of risk factors on outcomes can be calculated by the inverse-variance weighted (IVW) method, based on summarized data from all the genetic variants [56]. Therefore, MR analysis can be performed based on GWAS-summarized data, despite the lack of individual data [57]. MR analysis was also performed, based on the associations estimated by the beta coefficient and SEs between SNPs and exposure; between SNPs and outcome can be obtained from separate GWAS-summarized public data, known as two-sample MR. One-sample MR analysis differs from two-sample MR analysis in that the association between genetic variants and exposure outcomes is measured from all individuals in one sample.

For this reason, a two-sample MR can achieve the effect of using a much larger sample size compared to analysis using a single sample and thus can estimate the effect with statistically higher precision. In addition, although the exposure of interest and the outcome were not simultaneously measured within one data, it is a research design that can be analyzed if there is separate measurement data for each factor. In other words, since MR can be conducted with two independent observational epidemiological studies and does not require a large-scale longitudinal epidemiological study, it is especially useful to evaluate the comorbidities of patient cohorts [27].

For this study, we used the two-sample MR methods to confirm the causal effect of RA on the risk of breast cancer using ‘Sample 1’ for the estimation of SNP-RA and SNP-confounders for horizontal pleiotropy, and ‘Sample 2’ for the estimation of SNP-breast cancer association (Figure 2). 

We used the PRIVW method to evaluate the effect of RA on breast cancer using genetic variants as IVs [56,58]. In this MR method, the Wald ratio for each of the IVs is calculated to combine the results by IVW meta-analysis. The causal effect of the exposure on the outcome can be estimated by the slope from this analysis.

Three assumptions should be satisfied in MR analysis: (1) the genetic variants considered as IVs should be strongly associated with exposure, (2) the genetic variants referred as to IVs should not be associated with any confounding factors, and (3) the genetic variants as IVs should only have an effect on the risk of the outcome via exposure.

To evaluate whether the MR analysis follows the above three assumptions, we used a maximum likelihood estimation with Cochran’s Q heterogeneity tests to evaluate the possibility of horizontal pleiotropy [59,60] and to estimate the possibility of the genetic variants working as confounds between RA and breast cancer. A funnel plot was used to assess horizontal pleiotropy. Visually, the symmetrical graph suggests that horizontal pleiotropy could be present. We used penalized robust MR-Egger regression for sensitivity analysis to estimate the causal inference and horizontal pleiotropy [59]. Results with a *p*-value below 0.05 were considered statistically significant, and the intercept term from penalized robust MR Egger regression with a *p*-value below 0.05 were considered statistically significant in horizontal pleiotropy. Statistical analyses were conducted using the R 3.6 (R Core Team, Vienna, Austria) statistical software and Mendelian randomization package [61].

## 5. Conclusions

Even though the two-sample MR method is useful to evaluate the association between exposure and outcome by excluding the effects of other confounders, the causal inference between RA and breast cancer remains unclear. Once large-scale GWAS of RA effects are publicly available, the same method can be used to examine RA causally related to breast cancer and the relationship between RA and breast cancer. Additional research should include GWAS targeting both RA and breast cancer conducted in large-scale patient cohorts such as a nationwide patient cohort, using health insurance claim data and MR analyses to evaluate the correlation between RA and other cancers using GWAS summary statistics.

## Figures and Tables

**Table 1:** Results of penalized robust inverse variance weighted (PRIVW) method for causal inference of Rheumatoid arthritis as risk factors for breast cancer risk in two-sample Mendelian randomization.

Data of Summary Statistics	SNPs, n	Beta (SE)	OR (95% CI)
BBJ	24	−0.051 (0.021)	0.95 (0.91–0.99)
BCAC	25	0.014 (0.005)	1.01 (1.00–1.03)
BRCA1 carriers from CIMBA	25	−0.042 (0.007)	0.96 (0.95–0.97)
BRCA2 carriers from CIMBA	25	0.066 (0.038)	1.07 (0.99–1.15)

Single nucleotide polymorphisms, SNPs; standard error, SE; odds ratio, OR; confidence interval, CI; BBJ, BioBank Japan; BCAC, Breast Cancer Association Consortium; CIMBA, Consortium of Investigators of Modifiers of BRCA1/2.

**Table 2:** MR Egger regression for estimating average pleiotropic effect across the genetic variants in the causal inference of rheumatoid arthritis as a risk factor on the risk of breast cancer in two-sample Mendelian randomization.

Data of Summary Statistics	SNPs, n	Beta (SE)	*p*-Value
BBJ	24	−0.016 (0.008)	0.04
BCAC	25	−0.002 (0.002)	0.31
BRCA1 carriers from CIMBA	25	0.004 (0.007)	0.59
BRCA2 carriers from CIMBA	25	−0.031 (0.007)	≤0.001

Single nucleotide polymorphisms, SNPs; standard error, SE; odds ratio, OR; confidence interval, CI; BBJ, BioBank Japan; BCAC, Breast Cancer Association Consortium; CIMBA, Consortium of Investigators of Modifiers of BRCA1/2.

## Supplementary Materials

The following are available online at https://www.mdpi.com/2072-6694/12/11/3272/s1. Figure S1: Two-sample mendelian randomization (MR) estimates the causal effect of rheumatoid arthritis (RA) on the risk of breast cancer using the penalized robust inverse variance weighted method. (A–D) are based on genome-wide association study (GWAS)-summary statistics for breast cancer in BBJ, BCAC, CIMBA-BRCA1 carriers, and CIMBA-BRCA2 carriers, respectively. Forest plots show the estimated causal change (*β_GX_*) in standard deviations (SDs) with 95% confidence intervals (CIs), Figure S2: Funnel plots showing symmetry that indicates the absence of heterogeneity due to horizontal pleiotropy using penalized robust inverse variance weighted method (A–D) are based on genome-wide association study (GWAS)-summary statistics for breast cancer in BBJ, BCAC, CIMBA-BRCA1 carriers, and CIMBA-BRCA2 carriers, respectively, Figure S3: Flow chart for selecting single nucleotide polymorphisms (SNPs) as instrumental variables (IVs) from genome-wide association studies (GWAS)-summary statistics for the two-sample mendelian randomization (MR) analysis in East Asian population, Figure S4: Flow chart for selecting single nucleotide polymorphisms (SNPs) as instrumental variables (IVs) from genome-wide association studies (GWAS)-summary statistics for the two-sample mendelian randomization (MR) analysis in European population, Table S1: Summary statistics of single nucleotide polymorphisms (SNPs) from six different genome-wide association studies (GWAS) targeting rheumatoid arthritis (RA) conducted in East Asian population and the summary statistics of those SNPs in BioBank Japan (BBJ) GWAS summary results, Table S2: Summary statistics for single nucleotide polymorphisms (SNPs)-breast cancer association from Breast Cancer Association Consortium (BCAC) and Consortium of Investigators of Modifiers of BRCA1/2 (CIMBA) GWAS studies, Table S3: Shared selected pleiotropic loci for single nucleotide polymorphisms (SNPs) associated with rheumatoid arthritis (RA) from six different genome-wide association studies (GWAS)
