# Supplementary material for: Causal Inference between Rheumatoid Arthritis and Breast Cancer in East Asian and European Population: A Two-Sample Mendelian Randomization
Source: Cancers (Basel). 2020 Nov 5;12(11):3272. doi: 10.3390/cancers12113272 (PMC7694331; doi:10.3390/cancers12113272)
Supplement: Supplementary file 1 [file cancers-12-03272-s001.pdf]

Supplementary Materials

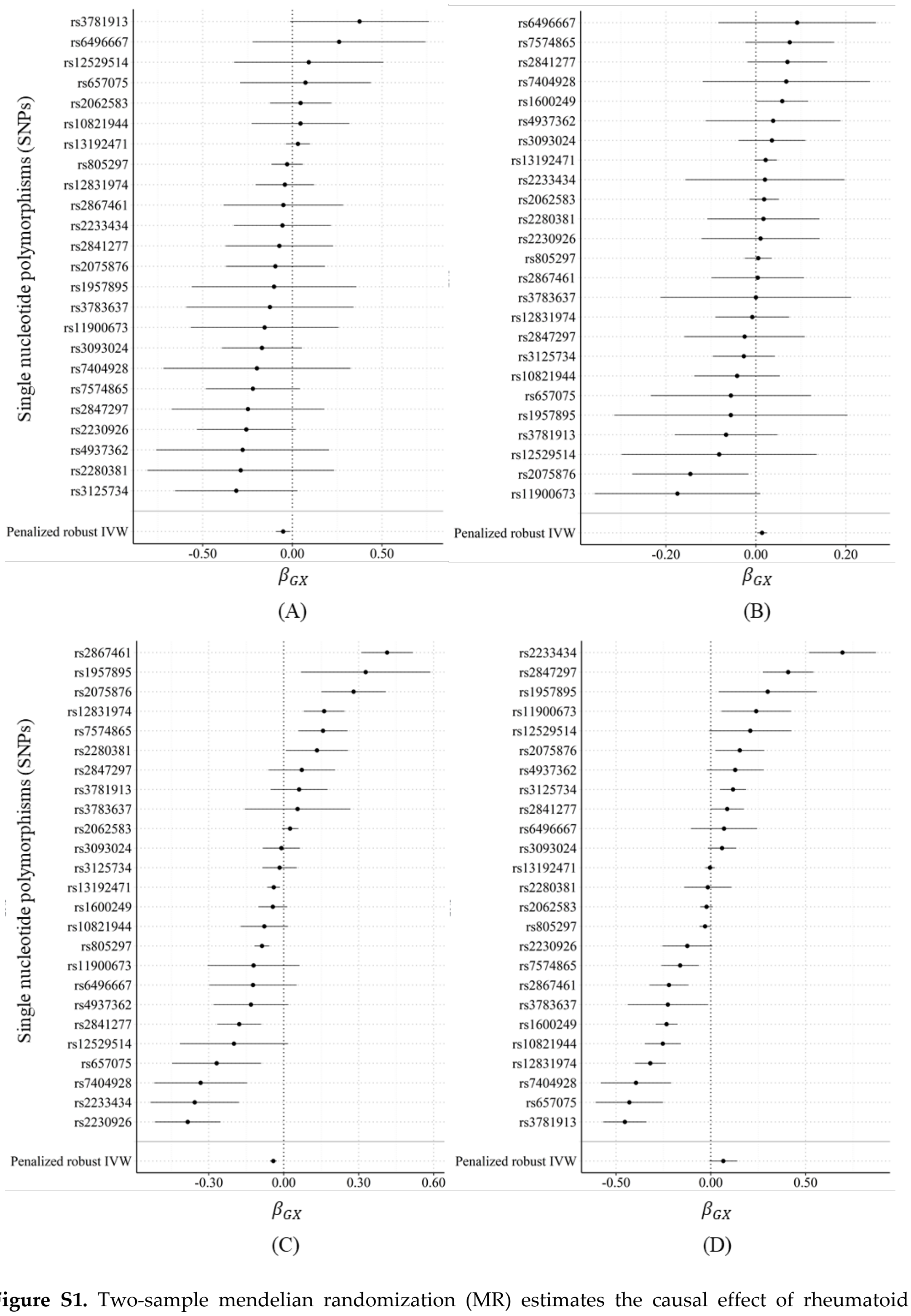

Figure S1. Two-sample mendelian randomization (MR) estimates the causal effect of rheumatoid arthritis (RA) on the risk of breast cancer using the penalized robust inverse variance weighted method. (**A**–**D**) are based on genome-wide association study (GWAS)-summary statistics for breast cancer in BBJ, BCAC, CIMBA-BRCA1 carriers, and CIMBA-BRCA2 carriers, respectively. Forest plots show the estimated causal change β_{GX} in standard deviations (SDs) with 95% confidence intervals (CIs).

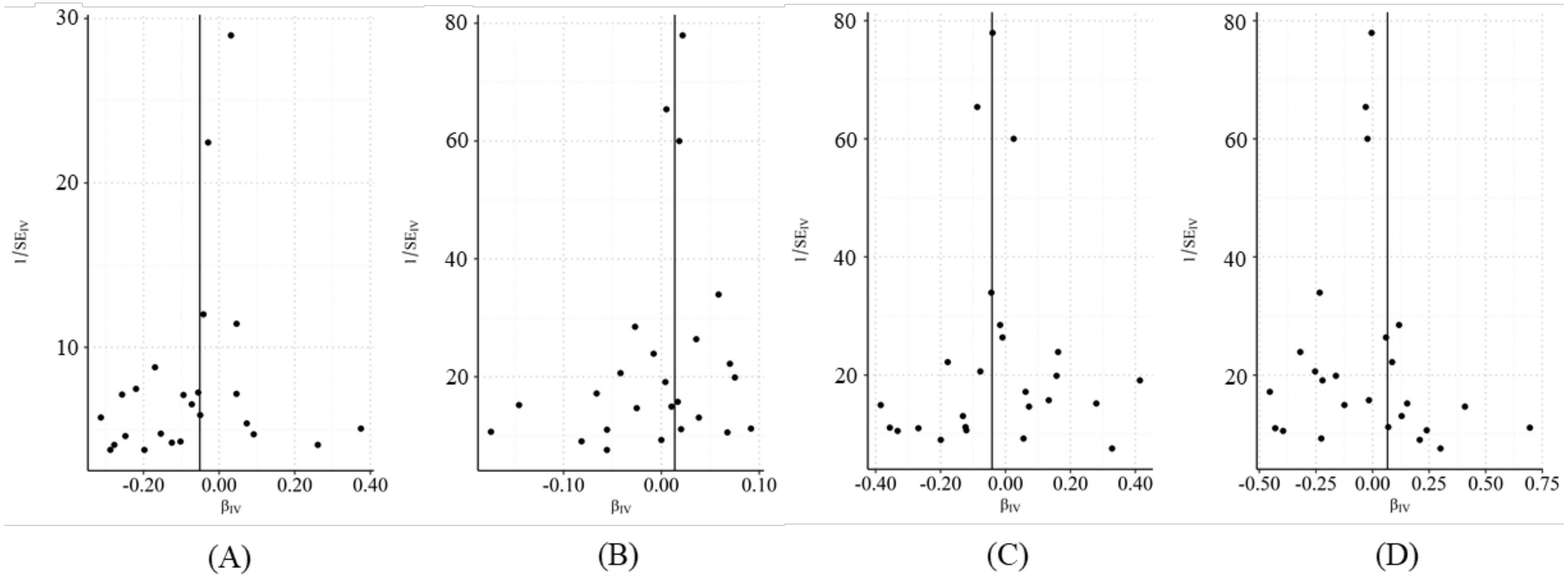

Figure S2. Funnel plots showing symmetry that indicates the absence of heterogeneity due to horizontal pleiotropy using penalized robust inverse variance weighted method (**A**–**D**) are based on genome-wide association study (GWAS)-summary statistics for breast cancer in BBJ, BCAC, CIMBA-BRCA1 carriers, and CIMBA-BRCA2 carriers, respectively.

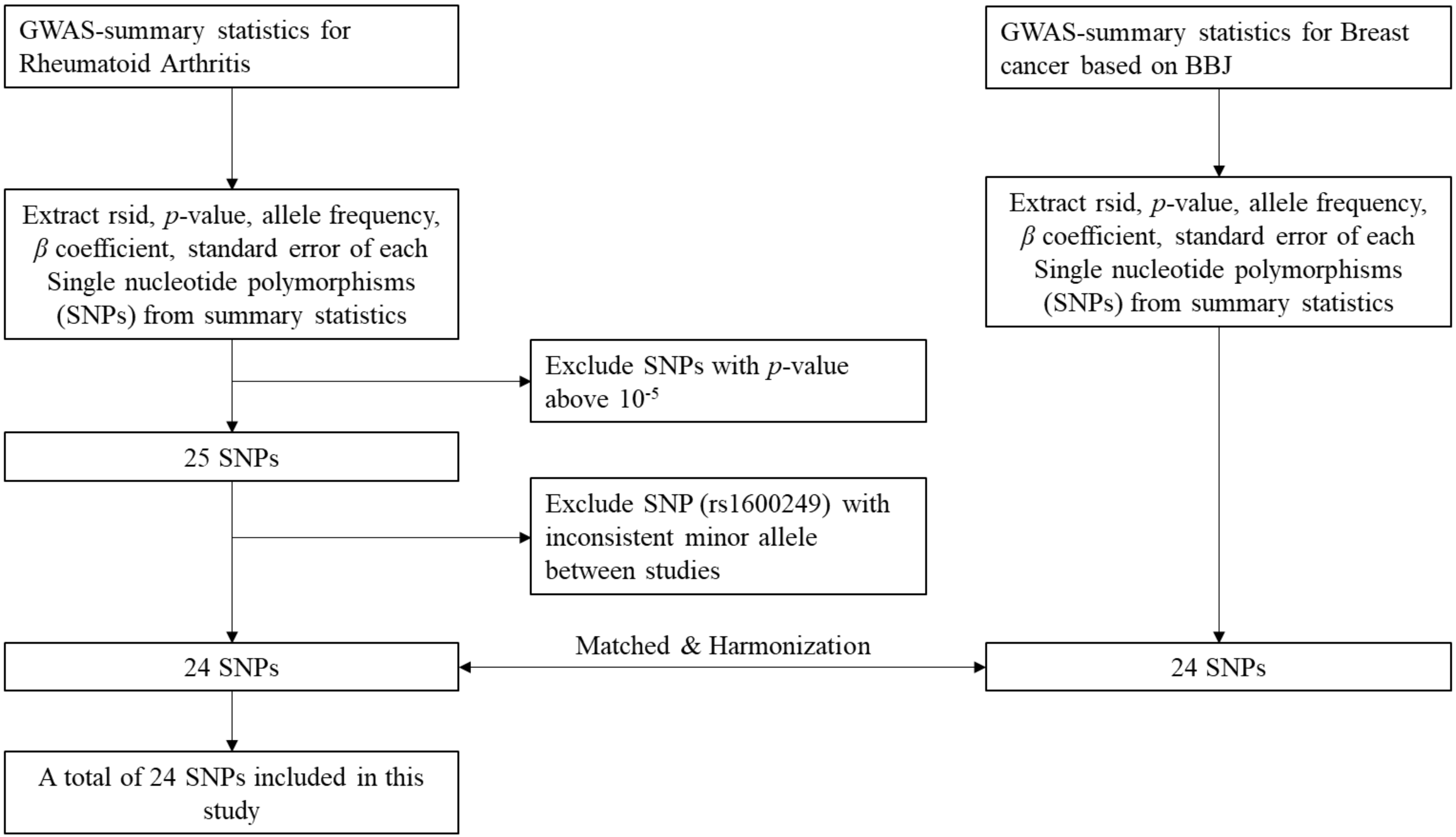

Figure S3. Flow chart for selecting single nucleotide polymorphisms (SNPs) as instrumental variables (IVs) from genome-wide association studies (GWAS)-summary statistics for the two-sample mendelian randomization (MR) analysis in East Asian population.

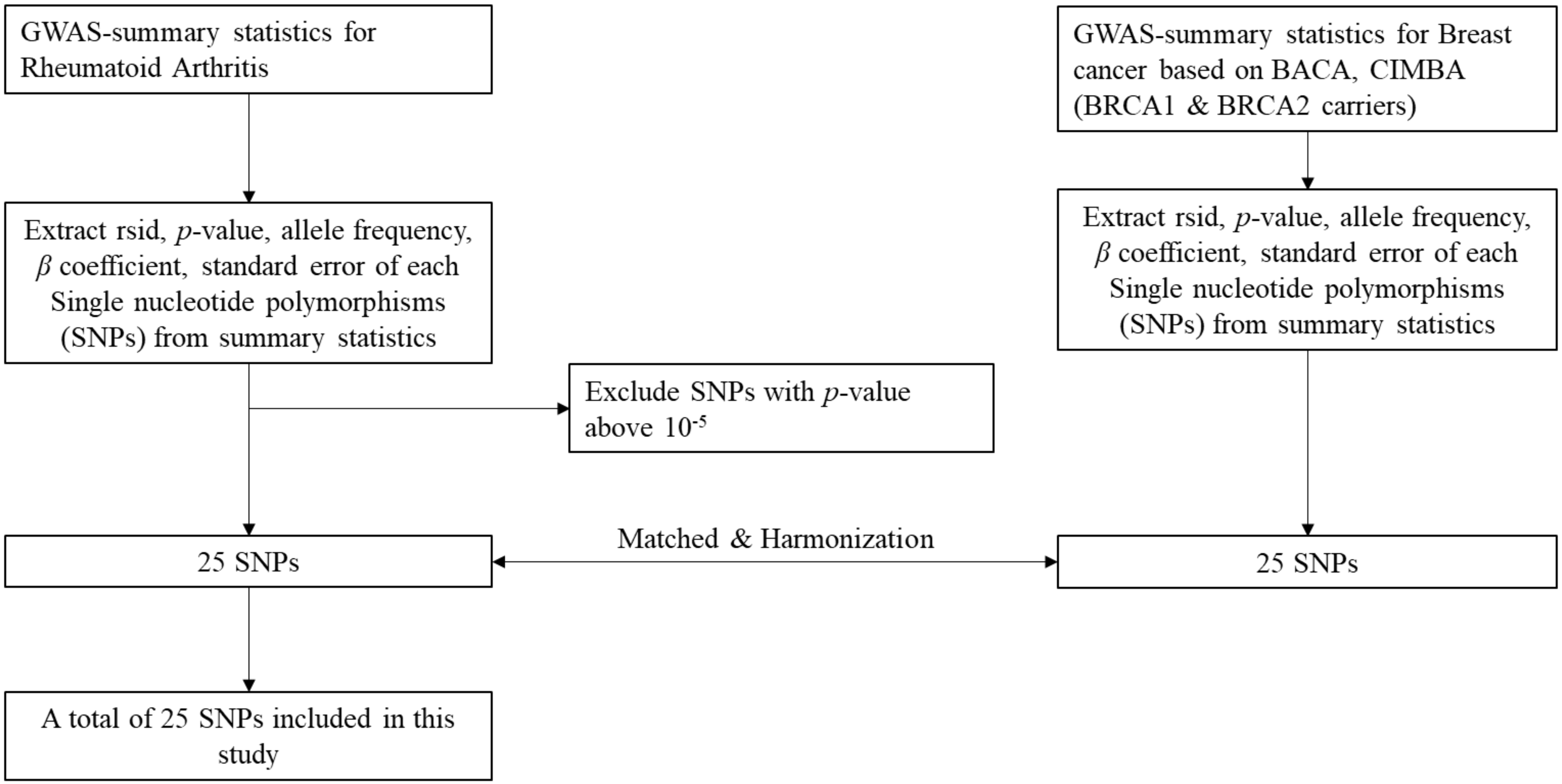

Figure S4. Flow chart for selecting single nucleotide polymorphisms (SNPs) as instrumental variables (IVs) from genome-wide association studies (GWAS)-summary statistics for the two-sample mendelian randomization (MR) analysis in European population.

Table S1. Summary statistics of single nucleotide polymorphisms (SNPs) from six different genome-wide association studies (GWAS) targeting rheumatoid arthritis (RA) conducted in East Asian population and the summary statistics of those SNPs in BioBank Japan (BBJ) GWAS summary results.

RA GWAS East Asian Studies						BBJ GWAS Summary Results			
**RS number**	**EA**	**EAF**	***p*-value**	**Beta**	**SE**	**RAF**	***p*-value**	Beta	SE
rs3093024	T	0.46	8×10^{-19}	0.1740	0.0210	0.47	0.14	−0.0295	0.0198
rs13192471	G	0.22	2×10^{-58}	0.6780	0.0422	0.24	0.36	0.0213	0.0234
rs7574865	T	0.33	2×10^{-6}	0.1570	0.0337	0.34	0.10	−0.0345	0.0210
rs2230926	C	0.07	2×10^{-6}	0.2700	0.0553	0.07	0.07	−0.0693	0.0379
rs12831974	C	0.42	6×10^{-6}	0.2390	0.0497	0.45	0.62	−0.0099	0.0199
rs2062583	G	0.10	2.16×10^{-6}	−0.4620	0.1024	0.06	0.60	−0.0213	0.0404
rs2075876	A	0.34	4×10^{-9}	0.1655	0.0253	0.34	0.50	−0.0156	0.0233
rs805297	A	0.36	3×10^{-10}	0.4447	0.0730	0.47	0.51	−0.0129	0.0198
rs2233434	G	0.21	5.8×10^{-19}	0.1740	0.0210	0.22	0.69	−0.0096	0.0240
rs3125734	T	0.11	5×10^{-9}	0.1823	0.0289	0.11	0.07	−0.0570	0.0318
rs11900673	T	0.28	1.1×10^{-8}	0.1044	0.0181	0.29	0.46	−0.0161	0.0220
rs2867461	A	0.44	1.2×10^{-12}	0.1222	0.0177	0.44	0.77	−0.0061	0.0208
rs657075	A	0.36	2.8×10^{-10}	0.1133	0.0135	0.36	0.69	0.0083	0.0211
rs12529514	C	0.14	2×10^{-8}	0.1310	0.0219	0.15	0.67	0.0120	0.0278
rs10821944	G	0.36	5.5×10^{-18}	0.1484	0.0173	0.37	0.74	0.0068	0.0207
rs3781913	T	0.69	5.8×10^{-10}	0.1133	0.0179	0.69	0.06	0.0425	0.0224
rs2841277	T	0.69	1.9×10^{-14}	0.1398	0.0174	0.69	0.64	−0.0101	0.0214
rs2847297	G	0.33	2.2×10^{-8}	0.0953	0.0182	0.35	0.25	−0.0236	0.0207
rs4937362	T	0.68	7.5×10^{-7}	0.0862	0.0184	0.68	0.26	−0.0239	0.0212
rs3783637	C	0.74	2×10^{-6}	0.0953	0.0182	0.75	0.60	−0.0119	0.0227
rs1957895	G	0.39	3.6×10^{-7}	0.0862	0.0184	0.39	0.66	−0.0088	0.0202
rs6496667	A	0.35	1.4×10^{-6}	0.0862	0.0184	0.35	0.29	0.0225	0.0212
rs7404928	T	0.62	4×10^{-6}	0.0770	0.0186	0.62	0.46	−0.0152	0.0205
rs2280381	T	0.84	2.4×10^{-6}	0.1133	0.0223	0.84	0.28	−0.0326	0.0301

EA, Effective Allele; EAF, Effective allele frequency; SE, Standard Error.

Table S2. Summary statistics for single nucleotide polymorphisms (SNPs)-breast cancer association from Breast Cancer Association Consortium (BCAC) and Consortium of Investigators of Modifiers of BRCA1/2 (CIMBA) GWAS studies.

BCAC GWAS Summary Results						CIMBA GWAS Summary Results				
**RS number**	**EA**	**EAF**	***p*-value**	**Beta**	**SE**	**Carriers**	**EAF**	***p*-value**	Beta	SE
rs3093024	T	0.45	0.35	0.0062	0.0066	BRCA1	0.93	0.02	−0.0016	0.0066
						BRCA2	0.68	0.03	0.0103	0.0066
rs13192471	G	0.15	0.09	0.0147	0.0087	BRCA1	0.30	0.03	−0.0271	0.0087
						BRCA2	0.93	0.03	−0.0028	0.0087
rs7574865	T	0.23	0.14	0.0118	0.0079	BRCA1	0.28	0.02	0.0247	0.0079
						BRCA2	0.39	0.03	−0.0254	0.0079
rs2230926	C	0.03	0.88	0.0028	0.0181	BRCA1	0.04	0.05	−0.1038	0.0181
						BRCA2	0.62	0.07	−0.0335	0.0181
rs1600249 [ǂ]	T	0.24	0.05	−0.0153	0.0077	BRCA1	0.60	0.02	0.0114	0.0077
						BRCA2	0.03	0.03	0.0610	0.0077
rs12831974	C	0.11	0.85	−0.0019	0.0100	BRCA1	0.18	0.03	0.03870	0.0100
						BRCA2	0.04	0.04	−0.0763	0.0100
rs2062583	G	0.25	0.27	−0.0084	0.0077	BRCA1	0.60	0.02	−0.0117	0.0077
						BRCA2	0.73	0.03	0.0104	0.0077

rs2075876	A	0.12	0.03	−0.0241	0.0109	BRCA1	0.13	0.03	0.0463	0.0109
						BRCA2	0.52	0.04	0.0253	0.0109
rs805297	A	0.31	0.73	0.0023	0.0068	BRCA1	0.05	0.02	−0.0387	0.0068
						BRCA2	0.60	0.03	−0.0134	0.0068
rs2233434	G	0.04	0.82	0.0035	0.0157	BRCA1	0.21	0.05	−0.0620	0.0157
						BRCA2	0.06	0.07	0.1209	0.0157
rs3125734	T	0.36	0.45	−0.0049	0.0064	BRCA1	0.87	0.02	−0.003	0.0064
						BRCA2	0.39	0.02	0.0214	0.0064
rs11900673	T	0.12	0.06	−0.0182	0.0098	BRCA1	0.66	0.03	−0.0126	0.0098
						BRCA2	0.50	0.04	0.0250	0.0098
rs2867461	A	0.36	0.93	0.0005	0.0064	BRCA1	0.01	0.02	0.0506	0.0064
						BRCA2	0.28	0.03	−0.0270	0.0064
rs657075	A	0.10	0.54	−0.0063	0.0103	BRCA1	0.32	0.03	−0.0304	0.0103
						BRCA2	0.22	0.04	−0.0487	0.0103
rs12529514	C	0.05	0.46	−0.0107	0.0145	BRCA1	0.54	0.04	−0.0261	0.0145
						BRCA2	0.63	0.06	0.0273	0.0145
rs10821944	G	0.28	0.39	−0.0062	0.0072	BRCA1	0.58	0.02	−0.0115	0.0072
						BRCA2	0.16	0.03	−0.0376	0.0072
rs3781913	T	0.56	0.25	−0.0075	0.0066	BRCA1	0.71	0.02	0.0070	0.0066
						BRCA2	0.04	0.02	−0.0514	0.0066
rs2841277	T	0.53	0.12	0.0098	0.0063	BRCA1	0.18	0.02	−0.0249	0.0063
						BRCA2	0.61	0.02	0.0121	0.0063
rs2847297	G	0.34	0.71	−0.0024	0.0065	BRCA1	0.72	0.02	0.0069	0.0065
						BRCA2	0.13	0.03	0.0389	0.0065
rs4937362	T	0.56	0.62	0.0033	0.0066	BRCA1	0.56	0.02	−0.0113	0.0066
						BRCA2	0.67	0.03	0.0111	0.0066
rs3783637	C	0.88	1.00	0.0000	0.0103	BRCA1	0.86	0.03	0.0053	0.0103
						BRCA2	0.58	0.04	−0.0216	0.0103
rs1957895	G	0.09	0.07	−0.0048	0.0114	BRCA1	0.38	0.03	0.0283	0.0114
						BRCA2	0.55	0.04	0.0259	0.0114
rs6496667	A	0.20	0.31	0.0079	0.0077	BRCA1	0.65	0.02	−0.0106	0.0077
						BRCA2	0.84	0.03	0.0060	0.0077
rs7404928	T	0.74	0.47	0.0052	0.0073	BRCA1	0.22	0.02	−0.0256	0.0073
						BRCA2	0.27	0.03	−0.0304	0.0073
rs2280381	T	0.63	0.79	0.0019	0.0072	BRCA1	0.45	0.02	0.0151	0.0072
						BRCA2	0.94	0.03	−0.0018	0.0072

EA, Effective Allele; EAF, Effective allele frequency; SE, Standard Error. ᵻ rs1600249 was removed in two-sample Mendelian randomization (MR) analysis based on East Asian population due to the inconsistency of minor allele frequency.

Table S3. Shared selected pleiotropic loci for single nucleotide polymorphisms (SNPs) associated with rheumatoid arthritis (RA) from six different genome-wide association studies (GWAS).

RS Number	Location	Gene Function	Mapped Gene	Mapped Phenotypes
rs3093024	6:167119305	Non-coding transcript exon variant	CCR6, Z94721.2, AL121935.1	Rheumatoid arthritis
rs13192471	6:32703326	Intergenic variant	MTCO3P1, HLA-DQB1	Rheumatoid arthritis, Non-melanoma skin carcinoma
rs7574865	2:191099907	Intron variant	STAT4	Rheumatoid arthritis, Systemic lupus erythematosus, Immune system disease, Systemic scleroderma, Hepatocellular carcinoma, Biliary liver cirrhosis, Sjogren syndrome, Autoimmune disease, Primary biliary cirrhosis
rs2230926	6:137874929	Missense variant	TNFAIP3	Rheumatoid arthritis, Systemic lupus erythematosus, Systemic scleroderma,
rs1600249	8:11502129	Intron variant	BLK	Rheumatoid arthritis
rs12831974	12:72330254	Intron variant	TRHDE	Rheumatoid arthritis
rs2062583	3:56932218	Intron variant	ARHGEF3	Rheumatoid arthritis
rs2075876	21:44289270	Non-coding transcript exon variant	AIRE	Rheumatoid arthritis
rs805297	6:31654829	Intron variant	APOM	Rheumatoid arthritis
rs2233434	6:44265183	Missense variant	NFKBIE	Rheumatoid arthritis
rs3125734	10:62198353	Missense variant	RTKN2	Rheumatoid arthritis
rs11900673	2:62225526	Intergenic variant	RN7SL51P, B3GNT2	Rheumatoid arthritis
rs2867461	4:78592061	Intron variant	ANXA3	Rheumatoid arthritis
rs657075	5:132094425	Regulatory region variant	AC063976.3, AC034216.1	Rheumatoid arthritis
rs12529514	6:14096427	Regulatory region variant	CD83, AL022396.1	Rheumatoid arthritis
rs10821944	10:62025330	3′ UTR variant	ARID5B	Rheumatoid arthritis, Blood urea nitrogen measurement, Glomerular filtration rate
rs3781913	11:72662452	Intron variant	PDE2A	Rheumatoid arthritis
rs2841277	14:104924668	Intergenic variant	PLD4, AL583810.3	Rheumatoid arthritis
rs2847297	18:12797695	Intron variant	PTPN2	Rheumatoid arthritis,

rs4937362	11:128622844	Intron variant	AP001122.1	Acute myeloid leukemia Rheumatoid arthritis, Neoplasm of mature B-cells
rs3783637	14:54881400	Intron variant	GCH1	Rheumatoid arthritis, Urinary metabolite measurement
rs1957895	14:61441614	Intron variant	PRKCH	Rheumatoid arthritis
rs6496667	15:90350436	Intergenic variant	AC018946.2, ZNF774	Rheumatoid arthritis
rs7404928	16:23877519	Intron variant	PRKCB	Rheumatoid arthritis, Primary biliary cirrhosis
rs2280381	16:85985027	Intron variant	AC092723.4	Rheumatoid arthritis

Mapped gene and phenotypes were reported in the GWAS catalog.
